# Reduced task-related quadriceps sEMG and limited functional transfer after 4-week low-load blood flow restriction training: a pilot randomized controlled study

**DOI:** 10.3389/fphys.2026.1785050

**Published:** 2026-03-25

**Authors:** Junwei Xia, Wonil Son, Tianqi Zhao, Ji-Kwang Ryu

**Affiliations:** 1 Department of Leisure and Sports, Kangwon National University, Samcheok, Republic of Korea; 2 Physical Education Measurement and Evaluation Lab, Department of Human Sports, College of Humanities, Social Science and Design Sports, Kangwon National University, Samcheok-si, Gangwon-do, Republic of Korea; 3 Department of Human Sports, College of Humanities, Social Science and Design Sports, Kangwon National University, Samcheok-si, Gangwon-do, Republic of Korea

**Keywords:** blood flow restriction, functional performance, isokinetic strength, neuromuscular efficiency, perceived exertion, surface electromyography

## Abstract

**Background:**

Low-load blood-flow restriction training (BFR-LIR) can enhance strength-related adaptations with reduced mechanical stress, but whether it promotes task-related neuromuscular economy (lower task-related sEMG amplitude for comparable mechanical output) and how it compares with high-intensity resistance training (HIR) remains unclear.

**Objective:**

To compare the effects of 4 weeks of BFR-LIR, HIR, and LIR on isokinetic knee extensor performance, task-related neuromuscular activation, functional performance, and perceived exertion in healthy young men.

**Methods:**

Twenty-four healthy young men were randomized (1:1:1) to a low-load blood-flow restriction training group (BFR-LIR; ∼30% baseline isokinetic peak torque with individualized cuff pressure set at 70% arterial occlusion pressure [AOP] using a 10-cm pneumatic cuff), a high-intensity resistance training group (HIR; 8–12 RM), or a low-intensity resistance training group (LIR; ∼30% baseline isokinetic peak torque). All groups trained three times per week for 4 weeks. Outcomes were assessed pre- and post-intervention (isokinetic knee extensor peak torque at 60°/s and 120°/s, quadriceps sEMG during standardized isokinetic testing, countermovement jump and standing long jump performance, and ratings of perceived exertion). Between-group effects were evaluated using one-way ANOVA on Pre–Post change scores (Δ) with Holm-adjusted *post hoc* comparisons, and effect sizes were reported. RPE was recorded immediately after the standardized isokinetic testing protocol (not during the jump tests).

**Results:**

Peak torque did not significantly change from pre-to post-intervention in any group at either velocity. Between-group comparisons of change scores (Δ) identified a significant group effect for Total Work at both 60°/s and 120°/s, driven by larger increases in HIR than LIR. Within-group Total Work increased at 60°/s in BFR-LIR and HIR, and at 120°/s in HIR. In BFR-LIR, Mean %MVC and dynamic RMS during isokinetic testing decreased at both velocities, whereas no consistent changes were observed in HIR or LIR. Hang time improved in HIR; standing long jump and RPE were unchanged across groups. No training-related adverse events were reported.

**Conclusion:**

Over 4 weeks, BFR-LIR was associated with small within-group improvements in isokinetic total work at 60°/s and reduced task-related quadriceps sEMG during isokinetic testing, whereas HIR showed the clearest improvement in hang time. Between-group differences in ΔsEMG were not significant, and changes did not translate to multi-joint jump performance over the short intervention period.

## 1 Introduction

Blood-flow restriction (BFR) training combines low-load resistance exercise with external cuff pressure to partially restrict arterial inflow and limit venous return, thereby increasing metabolic stress at relatively low mechanical loads (Patterson et al., 2019; Pearson and Hussain, 2015). This approach has attracted attention in both performance and rehabilitation contexts because low-load BFR training can elicit meaningful improvements in muscle strength and hypertrophy while reducing mechanical stress compared with traditional high-load resistance training (Slysz et al., 2016; Lixandrão et al., 2018; Grønfeldt et al., 2020). Consequently, BFR is increasingly considered as an option to preserve or restore muscular function when high loads are undesirable or not tolerated, such as during musculoskeletal rehabilitation or early return-to-sport phases (Hughes et al., 2017; Cognetti et al., 2022).

Despite the growing evidence base for morphological and strength adaptations, an important mechanistic question remains insufficiently resolved: whether BFR training improves “neuromuscular economy,” broadly defined as achieving the same or greater mechanical output with reduced neural activation requirements (Centner and Lauber, 2020). Surface electromyography (sEMG) is commonly used to quantify task-related neuromuscular activation, and acute BFR exercise can modify activation and fatigue profiles (de Queiros et al., 2021). However, sEMG amplitude is not a direct measure of “neural drive” and can be influenced by motor unit recruitment, firing behavior, muscle fiber conduction velocity, and signal cancellation. Therefore, reductions in EMG amplitude should be interpreted cautiously and ideally alongside mechanical outcomes and rigorous normalization procedures (Vigotsky et al., 2018; Martinez-Valdes et al., 2018).

Methodological heterogeneity—particularly cuff pressure prescription and cuff characteristics—may also contribute substantially to mixed neuromuscular findings. Contemporary recommendations emphasize prescribing pressure relative to limb/arterial occlusion pressure (LOP/AOP) rather than using fixed absolute pressures, because the same cuff pressure can produce different restriction levels across individuals (Patterson et al., 2019; Loenneke et al., 2012). In addition, LOP/AOP determination can vary with measurement posture, and lower-limb AOP reliability can differ across body positions, which has direct implications for reproducibility and between-study comparability (Hughes et al., 2018). Cuff width further modulates the pressure required to achieve a given restriction level and can influence training adaptations (Laurentino et al., 2016), while cuff design can alter acute hemodynamic and perceptual responses even under similar pressure targets (Mouser et al., 2017). Finally, safety considerations—including screening for contraindications and appropriate pressure/volume progression—remain central to BFR implementation (Loenneke et al., 2011; Patterson et al., 2019).

Another unresolved issue concerns the transfer of BFR-induced adaptations to functional performance. Because BFR protocols are frequently implemented using low loads and, in research settings, often emphasize isolated or single-joint actions, improvements in isolated strength or work capacity may not directly transfer to multi-joint, explosive tasks such as jumping that require intermuscular coordination and technique (Wortman et al., 2021). Although meta-analytic evidence suggests that both BFR and traditional high-load resistance training can improve strength outcomes, the magnitude and pattern of transfer to functional tasks may differ depending on training intensity, exercise selection, and testing specificity (Grønfeldt et al., 2020; Chang et al., 2023).

Therefore, the present study aimed to compare the effects of 4 weeks of (i) low-load resistance training with blood-flow restriction (BFR-LIR), (ii) high-intensity resistance training (HIR), and (iii) low-load resistance training without BFR (LIR) on isokinetic knee extensor performance, task-related neuromuscular activation, functional performance, and perceived exertion in healthy young men. We hypothesized that BFR-LIR would improve isokinetic performance compared with LIR and demonstrate a “neuromuscular economy” pattern (i.e., comparable or improved mechanical output with reduced task-related EMG amplitude), while HIR would yield robust mechanical improvements with different activation signatures (Centner and Lauber, 2020; Patterson et al., 2019).

## 2 Materials and methods

### 2.1 Participants

The study protocol was approved by the Institutional Review Board of Kangwon National University (KWNUIRB-2025-07-002-001). All participants received a detailed explanation of the study procedures, potential risks, and benefits, and provided written informed consent prior to participation. Twenty-four healthy, recreationally active male university students (age: 21.3 ± 1.7 years; height: 174.2 ± 5.9 cm; body mass: 69.8 ± 6.3 kg) were recruited. Inclusion criteria were the absence of musculoskeletal injury, cardiovascular or neurological disorders, and no prior resistance training experience. The sample size (n = 24; 8 per group) was determined *a priori* based on study feasibility and consistency with prior short-term BFR training trials using comparable isokinetic and sEMG outcomes. Participants were randomly assigned to one of three groups (n = 8 per group).

Because the sample size (N = 24) was feasibility-driven, we conducted an *a posteriori* sensitivity analysis to describe what between-group effects could be detected with this design. For a one-way between-group comparison (3 groups, α = 0.05, N = 24), the minimum detectable effect size at 80% power corresponds to Cohen’s f ≈ 0.68 (η^2^ ≈ 0.32), indicating that the study was primarily powered to detect large effects. Accordingly, non-significant findings should be interpreted cautiously and considered exploratory.

**FIGURE 1:**
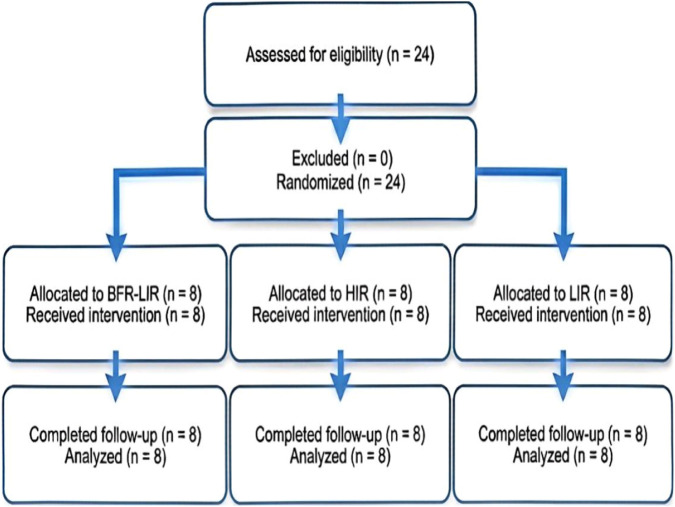
Participant flow diagram.

All randomized participants completed the 4-week intervention and pre/post assessments (n = 24; 8 per group) and were included in the analyses. No training-related adverse events were reported. Baseline characteristics were similar across groups (Table 1). Participant flow is shown in Figure 1.

**TABLE 1:** Baseline outcome measures by intervention group. Baseline between-group differences were examined using one-way ANOVA (p values shown) for descriptive transparency and were not used to infer the success of randomization.

Characteristic	BFR-LIR (n = 8)	HIR (n = 8)	LIR (n = 8)	p
Isokinetic knee extensor performance	Isokinetic knee extensor performance	Isokinetic knee extensor performance	Isokinetic knee extensor performance	
60°/s	60°/s	60°/s	60°/s	
Peak torque (N·m)	154.9 ± 22.7	174.9 ± 34.3	151.8 ± 31.2	0.265
Total work (J)	587.3 ± 102.9	591.3 ± 108.1	594.8 ± 96.6	0.989
120°/s	120°/s	120°/s	120°/s	
Peak torque (N·m)	132.7 ± 19.1	150.6 ± 36.6	130.3 ± 26.4	0.313
Total work (J)	557.5 ± 85.6	533.4 ± 92.2	560.5 ± 88.6	0.801

Quadriceps neuromuscular activation	Quadriceps neuromuscular activation	Quadriceps neuromuscular activation	Quadriceps neuromuscular activation	
60°/s	60°/s	60°/s	60°/s	
Dynamic RMS (µV)	148.3 ± 53.4	137.9 ± 69.0	169.9 ± 78.3	0.634
Mean %MVC (%)	48.3 ± 12.5	45.8 ± 14.9	46.9 ± 18.9	0.950
120°/s	120°/s	120°/s	120°/s	
Dynamic RMS (µV)	166.7 ± 61.4	154.7 ± 81.0	201.0 ± 101.2	0.521
Mean %MVC (%)	51.3 ± 14.7	48.7 ± 15.9	51.6 ± 25.4	0.947

Functional performance and perceived exertion	Functional performance and perceived exertion	Functional performance and perceived exertion	Functional performance and perceived exertion	
Hang time (s)	0.574 ± 0.067	0.548 ± 0.042	0.519 ± 0.034	0.110
Standing long jump (cm)	212.5 ± 11.7	200.5 ± 9.0	199.5 ± 17.1	0.110
RPE (6–20 scale)	14.5 ± 0.9	15.0 ± 1.6	15.2 ± 1.6	0.598

Values are presented as mean ± standard deviation (SD). Abbreviations: BFR-LIR, low-load resistance training with blood flow restriction; HIR, high-intensity resistance training; LIR, low-load resistance training without blood flow restriction; RMS, root mean square; MVC, maximal voluntary contraction; RPE, rating of perceived exertion. Baseline between-group differences were examined using one-way ANOVA, on individual baseline values (descriptive only).

### 2.2 Study design

This study used a randomized, parallel-group controlled design. After eligibility screening and a familiarization session, participants completed baseline testing (Pre), undertook a 4-week supervised knee-extension training program (3 sessions/week; 12 sessions total), and then repeated the same testing battery (Post). Participants were randomly allocated to one of three groups: low-load resistance training with blood flow restriction (BFR-LIR), high-intensity resistance training (HIR), or low-load resistance training without BFR (LIR). The allocation sequence was generated using a computer-based random-number generator with a 1:1:1 ratio. The Pre and Post testing procedures were identical and included Biodex isokinetic knee extension testing at 60°/s and 120°/s with concurrent sEMG, followed by functional performance tests and ratings of perceived exertion. The sample size (n = 24) was pre-specified based on feasibility (supervised training capacity and recruitment within the academic term) rather than an *a priori* power calculation for between-group effectiveness.

Randomization occurred after completion of baseline testing. Participants were enrolled and assigned to groups according to a computer-generated random sequence (1:1:1) prepared in advance. Allocation concealment was maintained using sequentially numbered, opaque, sealed envelopes (SNOSE). Each envelope was opened only after baseline assessments were completed.

Due to the nature of the intervention, participants and training supervisors could not be blinded to group allocation. However, all outcome assessments (isokinetic testing, functional tasks, and sEMG setup) were conducted by an assessor blinded to group allocation. To minimize detection and analytic bias, outcome assessments followed standardized procedures, sEMG processing was pre-specified, and statistical analyses were performed on coded datasets with group labels masked until primary analyses were finalized.

To reduce learning effects and acute fatigue confounding, all participants attended one familiarization session and were instructed to avoid strenuous lower-limb exercise for 48 h and caffeine and alcohol for 12 h before each testing visit. Testing was conducted at a similar time of day for each participant. Post testing was scheduled 48–72 h after the final training session.

For participants assigned to the BFR-LIR group, cuff pressure was individualized relative to arterial occlusion pressure (AOP). AOP was determined using Doppler ultrasound with participants in a seated position consistent with the Biodex knee extension testing posture, using a pneumatic cuff (width: 10 cm) placed proximally on the thigh. Training pressure was prescribed at 70% of the individually determined AOP and was intended to remain constant across training sessions. A predefined adjustment range (60%–80% AOP) was available only for tolerability (e.g., excessive discomfort or numbness/paresthesia), in which case pressure was reduced stepwise within the allowed range. No within-session or week-to-week pressure ramping strategy was implemented. This individualized approach was adopted to standardize the relative degree of blood flow restriction across participants (Patterson et al., 2019; Evin et al., 2021; de Queiros et al., 2021).

### 2.3 Training protocol summary

To improve clarity and replicability, a concise summary of the training prescriptions for each group is provided in Table 2.

**TABLE 2:** Four-week training protocol for the BFR-LIR, HIR, and LIR groups.

Grp	Load	Occl. pressure	Repetitions	Frequency	Rest interval	Duration	Notes
BFR-LIR	Low-load (∼30% baseline PT)	70% AOP; 10 cm cuff (proximal thigh)	30–15–15–15 per set	3×/week	30 s	4 weeks	70% AOP (60%–80% for comfort); continuous inflation during sets and rests; deflate after set 4
HIR	8–12RM (high-load)	None	4 × 8–12 reps	3×/week	60–90 s	4 weeks	8–12 RM corresponds to ∼70–85%1 RM (approx.). Loads adjusted session-to-session to stay within RM range (±2.5%–5%); 1RM not directly tested
LIR	Low-load (∼30% baseline PT)	None	30–15–15–15 per set	3×/week	30 s	4 weeks	Matched to BFR-LIR (same volume and cadence) without occlusion

AOP, arterial occlusion pressure; BFR, blood flow restriction; RM, repetition maximum; PT, peak torque; BFR-LIR, low-load BFR, training; HIR, high-intensity resistance training; LIR, low-load resistance training.

All training sessions were conducted under direct supervision three times per week for 4 weeks (12 sessions total) using unilateral knee extension exercise on the dominant leg. All sessions were performed on a seated knee-extension resistance machine, and individual seat/lever settings were recorded during familiarization and replicated across sessions. Each session consisted of a standardized warm-up (5–10 min cycling and dynamic stretches), the group-specific protocol (Table 2), and a brief cool-down. For the low-load conditions (BFR-LIR and LIR), the external load was set at approximately 30% of each participant’s baseline isokinetic peak torque and was maintained throughout the intervention to preserve a standardized mechanical demand across sessions. During familiarization, the knee-extension machine load was titrated in small increments to approximate the target low-load intensity (≈30% of baseline isokinetic peak torque). The selected absolute load (kg) was recorded and replicated across all subsequent sessions. During low-load sessions, the machine’s on-screen work-output display served as real-time feedback to support consistent effort while completing the pre-specified repetition scheme; investigators provided standardized verbal reminders when output visibly declined. For HIR, loads were adjusted session-to-session to ensure volitional failure within the 8–12 RM range: if a participant completed >12 repetitions with proper technique, the load was increased at the next session by the smallest achievable increment (typically ∼2.5–5%); if < 8 repetitions were achieved, the load was reduced by a comparable increment; otherwise, the load was maintained. Participants were instructed to avoid additional lower-limb resistance training during the intervention, and session attendance and completion were recorded.

### 2.4 Data collection

The cuff remained inflated throughout the four sets and the three 30-s inter-set rest intervals, resulting in an overall time under occlusion of approximately 5–7 min per session (depending on repetition duration). Immediately after the final set, the cuff was fully deflated to allow reperfusion. Participants were monitored throughout each session for excessive discomfort, numbness/paresthesia, and abnormal distal perfusion, and were instructed to report any adverse symptoms promptly.

#### 2.4.1 Training prescription and cuff inflation parameters

**FIGURE 2:**
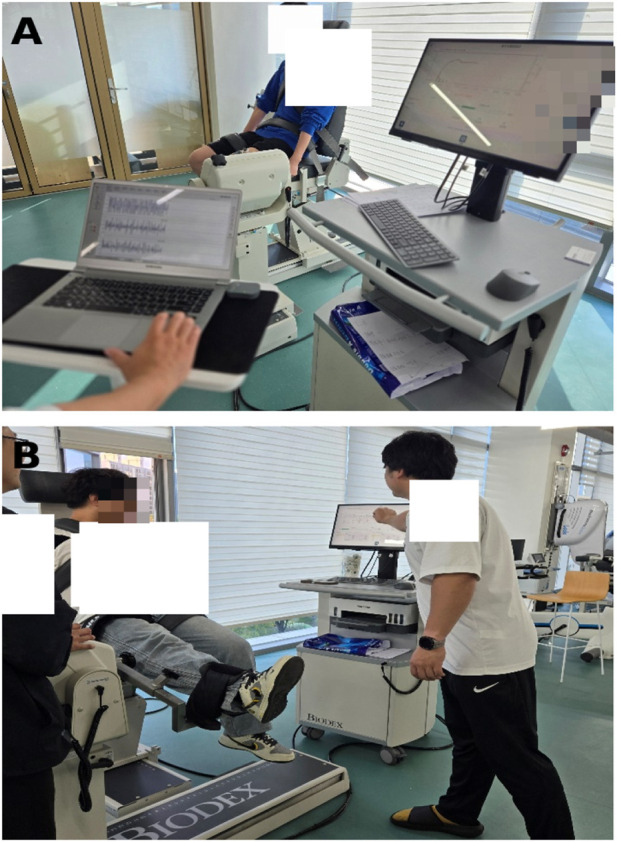
Representative experimental setup. **(A)** Shows the participant seated in the Biodex dynamometer with the pneumatic cuff applied (BFR-LIR) and concurrent sEMG acquisition. **(B)** Shows the Biodex-based isokinetic knee-extension testing setup.

BFR-LIR. Individualized blood flow restriction was implemented using a pneumatic cuff (Theratools, Guangzhou, China; width: 10 cm) positioned at the most proximal portion of the dominant thigh (Figure 2A). Training followed a low-load, high-repetition scheme (30–15–15–15 repetitions per set) with continuous cuff pressure maintained throughout the exercise and 30-s rest intervals.

To ensure precise pressure prescription, arterial occlusion pressure (AOP) was directly measured for each participant using a handheld Doppler ultrasound before the intervention. With participants in a seated position on the Biodex dynamometer (matching the training posture), the Doppler probe was used to detect the posterior tibial artery. Cuff pressure was gradually increased until the arterial pulse was no longer detectable, defining the individual AOP.

Training pressure was subsequently set at 70% of the measured AOP (Lixandrão et al., 2018), a protocol designed to maximize the adaptive stimulus while maintaining participant safety through individualized standardization (Patterson et al., 2019; de Queiros et al., 2021). No adverse events, such as excessive discomfort or paresthesia, were recorded during the study. All measurements were performed by the same investigator to ensure procedural consistency. The prescribed pressure was not progressively increased over the intervention.

HIR and LIR. HIR was performed using 4 sets of 8–12 repetition maximum (RM) loads. LIR and BFR-LIR utilized matched low loads, operationally defined as approximately 30% of each participant’s baseline isokinetic peak torque. This load was maintained throughout the 4-week intervention. Training frequency (3 sessions/week) was identical across groups; however, load prescription and inter-set rest intervals followed group-specific protocols (Table 2) (Patterson et al., 2019).

#### 2.4.2 Isokinetic strength

Isokinetic strength testing was performed using a Biodex System 4 Pro (Biodex Medical Systems, Shirley, NY, United States). Participants were seated and stabilized with trunk, pelvic, and thigh straps. The dynamometer axis was aligned with the lateral femoral epicondyle and the lever arm pad was secured proximal to the medial malleolus. The knee joint range of motion was set from 90° flexion to 0° extension (0° = full extension), and identical settings were used at Pre and Post. Gravity correction was applied before each test according to the manufacturer’s procedure. Following a standardized warm-up (5–10 min cycling and dynamic stretching) and several submaximal practice repetitions, participants performed maximal concentric knee extensions at 60°/s (5 repetitions) and 120°/s (10 repetitions), with a 2-min seated rest between velocities. The testing order was fixed (60°/s then 120°/s) and identical at Pre and Post. Peak Torque was defined as the highest torque recorded during the repetition set at each velocity, and Total Work was defined as the cumulative work across repetitions.

All images were acquired during the knee-extension testing sessions performed at 60°/s and 120°/s. For the BFR-LIR condition (Panel A), cuff pressure was individualized and set at 70% of arterial occlusion pressure (AOP) as described in the Methods. Isokinetic torque data from the Biodex system and sEMG data from the BTS system were recorded concurrently. Images were anonymized to prevent participant identification.

#### 2.4.3 Surface electromyography (sEMG)

Surface electromyography (sEMG) was recorded from the dominant-leg quadriceps muscles—vastus lateralis (VL), rectus femoris (RF), and vastus medialis (VM)—using a BTS system. Skin preparation involved shaving, light abrasion, and cleaning with alcohol to reduce impedance. Disposable Ag/AgCl electrodes (≈10 mm diameter) were placed with a 20 mm inter-electrode distance following SENIAM recommendations (Hermens et al., 2000); a reference electrode was placed over the tibial tuberosity.

Signals were sampled at 1,000 Hz, band-pass filtered (20–450 Hz), and notch-filtered at 60 Hz. Filtered signals were full-wave rectified and smoothed using a moving root-mean-square (RMS) window (50 ms). For each velocity, two indices were derived during the concentric knee-extension phase: (i) Mean %MVC, calculated as the mean rectified EMG amplitude expressed as a percentage of the session-specific MVC, and (ii) dynamic RMS (µV). Values were computed for each muscle and then averaged across VL, RF, and VM to provide a composite quadriceps index (Tables 6, 7).

Before isokinetic testing at each visit, participants performed three 3–5 s maximal isometric knee extensions on the Biodex at a fixed knee angle (60° knee flexion), separated by 60 s rest, with strong verbal encouragement. The highest EMG amplitude across attempts was used as the MVC reference for that visit. Task EMG at Pre was normalized to the Pre MVC, and task EMG at Post was normalized to the Post MVC.

#### 2.4.4 Functional performance and perceived exertion during isokinetic testing

Hang Time (s) was assessed as vertical flight time using the same timing system across visits. Prior to baseline testing, participants completed three practice countermovement jumps to familiarize with the procedure. During each test visit, participants then performed three maximal countermovement jumps with standardized instructions; the best attempt was retained, with >=60 s rest between attempts. Standing long jump (SLJ, cm) was assessed using a standardized metric tape. Participants performed three trials with consistent arm-swing and landing criteria; the best distance was retained, with >=60 s rest between trials. Perceived exertion (RPE; Borg 6-20) was recorded immediately after completion of the standardized isokinetic testing protocol at each visit (i.e., not during the jump tests) and was used as a secondary outcome rather than to prescribe or equate training intensity across groups (Borg, 1982; Borg, 1998). RPE was collected to characterize perceived exertion/readiness during the evaluation protocol and to aid interpretation when laboratory outcomes do not translate to changes in functional performance over a short intervention.

### 2.5 Statistical analysis

Statistical analyses were performed using IBM SPSS Statistics (version 27; IBM Corp., Armonk, NY, United States). Normality was assessed using the Shapiro–Wilk test (Shapiro and Wilk, 1965) and inspection of distributional plots. Baseline group differences were evaluated using one-way ANOVA for descriptive transparency (p values shown), but were not used to infer the success of randomization. Outcomes were analyzed separately for each velocity (60°/s and 120°/s).

Primary outcomes were isokinetic knee extensor peak torque and total work at 60°/s and 120°/s. Secondary outcomes included task-related quadriceps sEMG (Mean %MVC and Dynamic RMS), functional performance (hang time and standing long jump), and perceived exertion (RPE); secondary outcomes were treated as exploratory.

For primary between-group inference, Pre–Post change scores (Δ = Post − Pre) were compared among groups using one-way ANOVA. Significant omnibus tests were followed by Holm-adjusted pairwise comparisons (Holm, 1979). Between-group eff Between-group effect size was reported as eta-squared (η^2^).

Within-group Pre–Post changes were examined using paired-samples t-tests. To control the family-wise error across the three within-group tests for each outcome, Holm-adjusted p values are reported. Within-group effect sizes were calculated as Cohen’s d_av. Statistical significance was set at p < 0.05. Data are presented as mean ± SD unless otherwise stated, and mean differences are reported with 95% confidence intervals.

## 3 Results

### 3.1 Isokinetic knee extensor performance at 60°/s and 120°/s

Pre- and post-intervention descriptive values for isokinetic outcomes at 60°/s and 120°/s are summarized in Tables 3, 4, respectively.

**TABLE 3:** Isokinetic outcomes at 60°/s (Knee extension; dominant leg) Significance: *p < 0.05, **p < 0.01, ***p < 0.001.

Variable	Grp	Pre (Mean ± SD)	Post (Mean ± SD)	Mean diff [95%CI]	p	d_av	pHolm
Peak torque (N·m)	BFR-LIR	154.94 ± 22.68	162.07 ± 25.39	7.13 [−7.92, 22.19]	0.299	0.30	0.599
Peak torque (N·m)	HIR	174.87 ± 34.26	192.60 ± 38.36	17.73 [−7.56, 43.01]	0.141	0.49	0.424
Peak torque (N·m)	LIR	151.75 ± 31.24	151.34 ± 33.52	−0.41 [−14.79, 13.97]	0.948	−0.01	0.948
Total work (J)	BFR-LIR	587.31 ± 102.90	679.68 ± 117.07	92.37 [18.54, 166.19]	0.021	0.84	0.042*
Total work (J)	HIR	591.28 ± 108.13	712.91 ± 134.61	121.63 [67.21, 176.06]	0.001	1.00	0.003**
Total work (J)	LIR	594.76 ± 96.59	612.28 ± 105.04	17.51 [−45.85, 80.88]	0.534	0.17	0.534

Values are presented as mean ± SD. Mean Diff indicates the within-group change (Post − Pre) with 95% confidence intervals (95% CI). Within-group comparisons were performed using paired t-tests. Holm-adjusted p values (pHolm) are reported to correct for multiple comparisons across the three groups for each outcome. Effect size is reported as paired Cohen’s d_av (d_av). Statistical significance was set at p < 0.05.

**TABLE 4:** Isokinetic outcomes at 120°/s (Knee extension; dominant leg) Significance: *p < 0.05, **p < 0.01, ***p < 0.001.

Variable	Grp	Pre (Mean ± SD)	Post (Mean ± SD)	Mean diff [95%CI]	p	d_av	pHolm
Peak torque (N·m)	BFR-LIR	132.67 ± 19.06	138.38 ± 21.46	5.71 [−8.57, 19.99]	0.376	0.28	0.751
Peak torque (N·m)	HIR	150.57 ± 36.60	164.91 ± 37.72	14.34 [−6.60, 35.28]	0.149	0.39	0.448
Peak torque (N·m)	LIR	130.30 ± 26.43	128.04 ± 27.90	−2.26 [−16.45, 11.93]	0.717	−0.08	0.751
Total work (J)	BFR-LIR	557.46 ± 85.61	652.70 ± 110.73	95.24 [10.54, 179.94]	0.033	0.96	0.065
Total work (J)	HIR	533.41 ± 92.20	657.32 ± 159.53	123.91 [48.56, 199.25]	0.006	0.95	0.018*
Total work (J)	LIR	560.52 ± 88.61	564.47 ± 99.89	3.95 [−49.75, 57.66]	0.867	0.04	0.867

Values are presented as mean ± SD. Mean Diff indicates the within-group change (Post − Pre) with 95% confidence intervals (95% CI). Within-group comparisons were performed using paired t-tests. Holm-adjusted p values (pHolm) are reported to correct for multiple comparisons across the three groups for each outcome. Effect size is reported as paired Cohen’s d_av (d_av). Statistical significance was set at p < 0.05.

Peak torque was largely maintained across groups at both velocities. Between-group comparisons of change scores (Δ) identified group effects for Total Work at both 60°/s and 120°/s, driven by larger increases in HIR compared with LIR (Tables 3–5). Change scores for Peak Torque did not differ among groups (Table 5).

**TABLE 5:** Between-group comparisons of Pre–Post change scores (Δ) in primary isokinetic outcomes Significance: *p < 0.05, **p < 0.01, ***p < 0.001.

Outcome (velocity)	Δ BFR-LIR (Mean ± SD)	Δ HIR (Mean ± SD)	Δ LIR (Mean ± SD)	F (2,21)	p	η^2^	Post hoc (Holm)
Peak torque (N·m) (60°/s)	7.13 ± 18.01	17.73 ± 30.24	−0.41 ± 17.20	1.30	0.294	0.11	n.s.
Total work (J) (60°/s)	92.37 ± 88.31	121.63 ± 65.10	17.51 ± 75.79	3.89	0.036*	0.27	HIR > LIR (p = 0.032)
Peak torque (N·m) (120°/s)	5.71 ± 17.08	14.34 ± 25.05	−2.26 ± 16.97	1.37	0.276	0.12	n.s.
Total work (J) (120°/s)	95.24 ± 101.31	123.91 ± 90.12	3.95 ± 64.24	4.18	0.030*	0.28	HIR > LIR (p = 0.028)

Δ indicates within-subject change scores (Post − Pre). One-way ANOVA was applied to Δ for each outcome; significant effects were followed by Holm-adjusted pairwise comparisons. η^2^ denotes eta-squared.

Between-group comparisons of Pre–Post change scores (Δ) for the primary isokinetic outcomes are reported in Table 5.

### 3.2 Neuromuscular activation during isokinetic testing at 60°/s and 120°/s

Within-group pre–post comparisons of sEMG outcomes at 60°/s and 120°/s are presented in Tables 6, 7, respectively.

**TABLE 6:** sEMG Outcomes at 60°/s: Mean %MVC and Dynamic RMS—Within-Group Pre–Post Comparisons Significance: *p < 0.05, **p < 0.01, ***p < 0.001.

Variable	Grp	Pre (Mean ± SD)	Post (Mean ± SD)	Mean diff [95% CI]	p	d_av	pHolm
Mean %MVC (%)	BFR-LIR	48.27 ± 12.54	28.63 ± 8.00	−19.64 [−27.67, −11.61]	0.001	−1.87	0.002**
Mean %MVC (%)	HIR	45.78 ± 14.93	34.65 ± 10.15	−11.13 [−24.19, 1.93]	0.084	−0.87	0.167
Mean %MVC (%)	LIR	46.87 ± 18.91	40.00 ± 10.10	−6.87 [−23.04, 9.30]	0.349	−0.45	0.349
Dynamic RMS (µV)	BFR-LIR	148.25 ± 53.44	104.69 ± 43.75	−43.56 [−75.50, −11.61]	0.015	−0.89	0.044*
Dynamic RMS (µV)	HIR	137.86 ± 68.96	99.69 ± 44.81	−38.16 [−87.85, 11.53]	0.112	−0.66	0.224
Dynamic RMS (µV)	LIR	169.93 ± 78.29	157.71 ± 79.35	−12.21 [−68.72, 44.29]	0.625	−0.15	0.625

Values are presented as mean ± SD. Mean %MVC represents the average EMG amplitude normalized to maximal voluntary contraction (MVC) as described in the Methods during the 60°/s task, and Dynamic RMS represents the root mean square (RMS) amplitude of the dynamic EMG signal (μV). Mean Diff indicates the within-group change (Post − Pre) with 95% confidence intervals (95% CI). Within-group comparisons were performed using paired t-tests. Holm-adjusted p values (pHolm) are reported to correct for multiple comparisons across the three groups for each EMG outcome. Effect size is reported as paired Cohen’s d_av (d_av). Statistical significance was set at p < 0.05.

**TABLE 7:** sEMG Outcomes at 120°/s: Mean %MVC and Dynamic RMS—Within-Group Pre–Post Comparisons Significance: *p < 0.05, **p < 0.01, ***p < 0.001.

Variable	Grp	Pre (Mean ± SD)	Post (Mean ± SD)	Mean diff [95%CI]	p	d_av	pHolm
Mean %MVC (%)	BFR-LIR	51.25 ± 14.70	30.00 ± 9.51	−21.26 [−30.71, −11.80]	0.001	−1.72	0.003**
Mean %MVC (%)	HIR	48.73 ± 15.89	36.37 ± 11.60	−12.36 [−25.33, 0.61]	0.059	−0.89	0.118
Mean %MVC (%)	LIR	51.61 ± 25.37	42.18 ± 11.30	−9.44 [−28.90, 10.02]	0.289	−0.48	0.289
Dynamic RMS (µV)	BFR-LIR	166.73 ± 61.39	116.71 ± 49.36	−50.02 [−82.93, −17.12]	0.009	−0.90	0.026*
Dynamic RMS (µV)	HIR	154.70 ± 81.02	111.59 ± 52.36	−43.11 [−98.20, 11.98]	0.107	−0.63	0.213
Dynamic RMS (µV)	LIR	200.99 ± 101.22	177.51 ± 98.82	−23.48 [−95.21, 48.25]	0.464	−0.23	0.464

Values are presented as mean ± SD. Mean %MVC represents the average EMG amplitude normalized to maximal voluntary contraction (MVC) as described in the Methods, and Dynamic RMS represents the root mean square (RMS) amplitude of the dynamic EMG signal (µV). Mean Diff indicates the within-group change (Post − Pre) with 95% confidence intervals (95% CI). Within-group comparisons were performed using paired t-tests. Holm-adjusted p values (pHolm) are reported to correct for multiple comparisons across the three groups for each EMG outcome. Effect size is reported as paired Cohen’s d_av. Statistical significance was set at p < 0.05.

Between-group comparisons of change scores (Δ) did not identify significant group effects for Mean %MVC or Dynamic RMS at either 60°/s or 120°/s (Table 8). Within-group analyses showed significant reductions in BFR-LIR for both Mean %MVC and Dynamic RMS at both velocities, whereas HIR and LIR did not demonstrate Holm-adjusted significant changes (Tables 6, 7).

**TABLE 8:** Between-group comparisons of Pre–Post change scores (Δ) in sEMG and secondary outcomes Significance: *p < 0.05, **p < 0.01, ***p < 0.001.

Outcome	Δ BFR-LIR (Mean ± SD)	Δ HIR (Mean ± SD)	Δ LIR (Mean ± SD)	F (2,21)	p	η^2^	Post hoc (Holm)
Mean %MVC (%) (60°/s)	−19.64 ± 9.61	−11.13 ± 15.62	−6.87 ± 19.34	1.43	0.262	0.12	n.s.
Dynamic RMS (µV) (60°/s)	−43.56 ± 38.21	−38.16 ± 59.44	−12.21 ± 67.59	0.71	0.505	0.06	n.s.
Mean %MVC (%) (120°/s)	−21.26 ± 11.31	−12.36 ± 15.51	−9.44 ± 23.28	1.00	0.385	0.09	n.s.
Dynamic RMS (µV) (120°/s)	−50.02 ± 39.36	−43.11 ± 65.90	−23.48 ± 85.80	0.34	0.713	0.03	n.s.
Hang time (s)	0.024 ± 0.025	0.073 ± 0.038	0.011 ± 0.039	7.08	0.004**	0.40	HIR > BFR-LIR (p = 0.020); HIR > LIR (p = 0.020)
Standing long jump (cm)	5.79 ± 8.47	11.06 ± 13.86	3.79 ± 12.43	0.81	0.459	0.07	n.s.
RPE (6–20)	−0.38 ± 1.29	−0.46 ± 0.99	−0.10 ± 1.32	0.20	0.823	0.02	n.s.

For sEMG and secondary outcomes, Δ indicates within-subject change scores (Post − Pre). One-way ANOVA compared change scores among groups. Significant omnibus tests were followed by Holm-adjusted pairwise comparisons. Negative Δ indicates reductions in sEMG and RPE. η^2^ denotes eta-squared.

### 3.3 Secondary outcomes: functional performance and perceived exertion during isokinetic testing

Between-group comparisons of change scores (Δ) showed a significant group effect for hang time (F (2,21) = 7.08, p = 0.004, η^2^ = 0.40), with greater improvements in HIR compared with BFR-LIR and LIR (Holm-adjusted p = 0.020 for both; Table 8). Change scores for standing long jump and RPE did not differ among groups (Table 8). Within-group analyses were consistent with these patterns, showing a significant increase in hang time after HIR (Table 9).

**TABLE 9:** Changes in functional performance and perceived exertion during isokinetic testing Significance: *p < 0.05, **p < 0.01, ***p < 0.001.

Outcome	Grp	Pre (Mean ± SD)	Post (Mean ± SD)	Mean diff [95% CI]	pHolm	d_av
Hang time (s)	BFR-LIR	0.574 ± 0.067	0.599 ± 0.079	0.024 [0.004, 0.045]	0.053	0.33
	HIR	0.548 ± 0.042	0.621 ± 0.064	0.073 [0.041, 0.105]	0.003**	1.34
	LIR	0.519 ± 0.034	0.530 ± 0.045	0.011 [−0.022, 0.044]	0.465	0.27
Standing long jump (cm)	BFR-LIR	212.45 ± 11.70	218.24 ± 10.37	5.79 [−1.30, 12.87]	0.189	0.52
	HIR	200.53 ± 8.96	211.59 ± 17.40	11.06 [−0.52, 22.65]	0.176	0.80
	LIR	199.50 ± 17.10	203.29 ± 19.17	3.79 [−6.61, 14.18]	0.418	0.21
RPE (6–20)	BFR-LIR	14.53 ± 0.91	14.15 ± 1.24	−0.38 [−1.45, 0.70]	0.870	−0.35
	HIR	14.99 ± 1.60	14.52 ± 1.34	−0.46 [−1.29, 0.37]	0.689	−0.31
	LIR	15.20 ± 1.57	15.10 ± 0.81	−0.10 [−1.20, 1.00]	0.870	−0.08

Values are presented as mean ± SD. Mean Diff indicates the within-group change (Post - Pre) with 95% confidence intervals (95% CI). Within-group comparisons were performed using paired t-tests. Holm-adjusted p values (pHolm) are reported to correct for multiple comparisons across the three groups for each outcome. Effect size is reported as paired Cohen’s d_av. RPE (Borg 6-20) was recorded immediately after completion of the standardized isokinetic testing protocol. Statistical significance was set at p < 0.05.

## 4 Discussion

### 4.1 Principal findings

This randomized, parallel-group study examined baseline (Pre) and 4-week (Post) changes in Biodex isokinetic knee extensor performance at 60°/s and 120°/s (peak torque and total work), concurrent task-related quadriceps sEMG during the same tests (Mean %MVC and Dynamic RMS), and functional performance. Between-group comparisons of change scores identified larger increases in total work after HIR than LIR, whereas change scores in peak torque did not differ among groups. Within-group, BFR-LIR showed increased total work at 60°/s (with a similar but non-significant trend at 120°/s) alongside reductions in task-related sEMG amplitude at both velocities; however, between-group differences in sEMG change scores were not statistically significant. Functional transfer was limited, with a significant between-group effect for hang time favoring HIR and no between-group differences for standing long jump or RPE.

HIR demonstrated numerically larger improvements in several isokinetic outcomes (e.g., peak torque and total work), but these changes did not reach conventional statistical significance in the present sample. Given the pilot sample size and the magnitude of the observed effect sizes, this pattern is compatible with limited statistical power (Type II error). Because post-testing occurred shortly after the final training session, transient residual fatigue or incomplete recovery may have attenuated maximal performance; however, RPE recorded after the standardized isokinetic testing protocol remained largely stable, so this interpretation should be considered tentative. Finally, the intervention employed isotonic resistance exercise whereas the primary strength outcomes were assessed isokinetically; such mode mismatch may reduce apparent transfer from training to testing.

### 4.2 Interpreting task-related “neuromuscular economy” from sEMG

In the present study, neuromuscular economy was operationally defined as maintaining or increasing isokinetic output at a fixed velocity while exhibiting lower task-related sEMG amplitude. This interpretation should be treated cautiously because sEMG amplitude is influenced by non-neural factors (e.g., electrode-skin impedance, subcutaneous tissue, and muscle fiber conduction properties) and does not uniquely quantify neural drive (Vigotsky et al., 2018; Martinez-Valdes et al., 2018). Nevertheless, under a standardized protocol (fixed task, electrode placement, and %MVC normalization), these observations should be interpreted cautiously. Because task EMG was normalized to session-specific MVC, changes in %MVC may partly reflect changes in MVC rather than a direct change in neural drive; therefore, we also considered the concurrent reductions in unnormalized dynamic RMS when interpreting task-related activation, particularly when between-group differences were not statistically significant. The consistent post-intervention reductions in Mean %MVC and dynamic RMS after BFR-LIR, alongside largely preserved peak torque, are compatible with reduced activation requirements for the same mechanical task. Such a pattern could arise from multiple mechanisms, including altered motor unit recruitment strategies, reduced unnecessary coactivation, and/or peripheral adaptations that improve contractile efficiency under hypoxic/metabolic stress (Sale, 1988; Carolan and Cafarelli, 1992; Aagaard et al., 2002; Pearson and Hussain, 2015).

### 4.3 Functional transfer and task specificity

Transfer to multi-joint functional tasks was limited and task-dependent. Hang time improved after HIR, whereas BFR-LIR showed only a small change and standing long jump performance did not clearly improve. These findings suggest that, over a 4-week period, adaptations observed in a constrained single-joint isokinetic task may not robustly generalize to jump performance, which depends on rapid force development, inter-segmental coordination, and technique (Aagaard et al., 2002; Maffiuletti et al., 2016; Huang et al., 2024).

Mechanistically, BFRT may elicit early neuromuscular adaptations through heightened metabolic stress and afferent feedback (group III/IV), which can augment central motor drive and facilitate recruitment of higher-threshold motor units at low external loads (Patterson et al., 2019; Centner and Lauber, 2020). Early resistance training studies suggest that measurable neuromuscular adaptations can emerge within ∼1–2 weeks (e.g., Jenkins et al., 2016), whereas transfer to explosive multi-joint tasks may lag due to task specificity and coordination demands (Aagaard et al., 2002; Maffiuletti et al., 2016). Moreover, BFRT has been proposed to increase fast-twitch fiber involvement at low loads, although clear fiber-type transitions likely require longer exposure. Therefore, the selected functional tests may have lacked sensitivity to detect subtle short-term neuromuscular changes over a 4-week period, particularly without jump-specific training.

### 4.4 Perceived exertion and training economy

Ratings of perceived exertion (RPE; Borg 6–20), recorded immediately after completion of the standardized isokinetic testing protocol at each visit, did not change meaningfully across the 4-week intervention, suggesting that perceived effort for the evaluation protocol remained stable (Borg, 1982; Borg, 1998).

### 4.5 BFR effectiveness and the role of individualized pressure

Strength-related benefits of BFR training depend strongly on how cuff pressure is prescribed. In the present study, arterial occlusion pressure (AOP) was measured with Doppler ultrasound and 70% AOP was applied using a 10 cm cuff, aligning with recommendations to standardize the relative restriction stimulus across individuals (Patterson et al., 2019). Given that cuff width, body position, and participant characteristics influence occlusion pressure, individualized AOP-based prescription is generally preferable to fixed absolute pressures (Hughes et al., 2018; Evin et al., 2021). In the present study, 70% AOP was applied using a 10-cm cuff and was maintained during the short inter-set rest intervals to maximize metabolic stress at very low loading. Notably, the cumulative time under occlusion per session was limited (approximately 5–7 min), and no adverse events were reported. Nonetheless, this combination represents a relatively high occlusive stimulus, and future studies should examine pressure and inflation dose–response relationships (e.g., 40%–60% vs. 80% AOP; intermittent vs. continuous inflation) to optimize the trade-off between stimulus, discomfort, and safety. Recent evidence suggests that higher relative AOP can increase muscle damage and perceptual strain in some contexts (Happ et al., 2025), and that acute responses of EMG, blood lactate, and RPE vary across AOP levels (Zhu et al., 2025), supporting the need for dose–response work on pressure and inflation strategies.

### 4.6 Limitations and future directions

Several limitations warrant consideration. First, this pilot study (n = 8 per group) was not powered to detect small-to-moderate between-group differences; therefore, non-significant findings—particularly for HIR—should be interpreted cautiously and in conjunction with effect sizes and confidence intervals. Second, sEMG was recorded only from quadriceps muscles; without antagonist hamstring sEMG, we cannot exclude changes in antagonist co-activation as a contributor to the reduced agonist activation observed at a given net torque. Third, surface EMG amplitude provides only a surrogate index of neural drive and can be influenced by peripheral factors (e.g., changes in action potential properties and amplitude cancellation), which limits mechanistic inference. Fourth, the intervention was short (4 weeks) and conducted in healthy young men, limiting generalizability to women, older adults, or clinical populations. Future studies should incorporate antagonist measurements and/or high-density EMG to clarify motor unit behavior, test dose–response effects of cuff pressure and inflation strategy (continuous vs. intermittent), and evaluate longer interventions and more task-specific training stimuli to improve functional transfer.

## 5 Conclusion

In conclusion, in this 4-week pilot randomized controlled study, BFR-LIR was associated with increased total work during 60°/s isokinetic knee-extension testing and reduced task-related quadriceps sEMG, suggesting a potential pattern of improved task-specific neuromuscular economy. Peak torque was largely maintained across groups, and functional transfer to jumping performance was limited (hang time improved only in HIR). Between-group Δ comparisons for sEMG were not significant, highlighting the exploratory nature of these findings and the potential need for longer and/or more task-specific training.

## Data Availability

The original contributions presented in the study are included in the article/supplementary material, further inquiries can be directed to the corresponding authors.
